# Species delimitation in asexual insects of economic importance: The case of black scale (*Parasaissetia nigra*), a cosmopolitan parthenogenetic pest scale insect

**DOI:** 10.1371/journal.pone.0175889

**Published:** 2017-05-01

**Authors:** Yen-Po Lin, Robert D. Edwards, Takumasa Kondo, Thomas L. Semple, Lyn G. Cook

**Affiliations:** 1 College of Life Science, Shanxi University, Taiyuan, Shanxi, China; 2 School of Biological Sciences, The University of Queensland, Brisbane, Queensland, Australia; 3 Research School of Biology, Division of Evolution, Ecology and Genetics, The Australian National University, Canberra, Australian Capital Territory, Australia; 4 Department of Botany, National Museum of Natural History, Smithsonian Institution, Washington DC, United States of America; 5 Corporación Colombiana de Investigación Agropecuaria (CORPOICA), Centro de Investigación Palmira, Valle del Cauca, Colombia; University of Innsbruck, AUSTRIA

## Abstract

Asexual lineages provide a challenge to species delimitation because species concepts either have little biological meaning for them or are arbitrary, since every individual is monophyletic and reproductively isolated from all other individuals. However, recognition and naming of asexual species is important to conservation and economic applications. Some scale insects are widespread and polyphagous pests of plants, and several species have been found to comprise cryptic species complexes. *Parasaissetia nigra* (Nietner, 1861) (Hemiptera: Coccidae) is a parthenogenetic, cosmopolitan and polyphagous pest that feeds on plant species from more than 80 families. Here, we implement multiple approaches to assess the species status of *P*. *nigra*, including coalescence-based analyses of mitochondrial and nuclear genes, and ecological niche modelling. Our results indicate that the sampled specimens of *P*. *nigra* should be considered to comprise at least two ecotypes (or "species") that are ecologically differentiated, particularly in relation to temperature and moisture. The presence of more than one ecotype under the current concept of *P*. *nigra* has implications for biosecurity because the geographic extent of each type is not fully known: some countries may currently have only one of the biotypes. Introduction of additional lineages could expand the geographic extent of damage by the pest in some countries.

## Introduction

Delineation of asexual lineages remains a challenge for taxonomists. The biological species concept [1] and other approaches that require consideration of mating (e.g. specific mate recognition species concept; [2]), gene pools (e.g. genetic species concept; [3–4]) and independent evolutionary trajectories (e.g. evolutionary species concept; [5]) are not meaningful for delimiting lineages in which every individual is reproductively isolated from every other and thus on its own evolutionary path. Other concepts, such as the phylogenetic species concept [6] and the mtDNA barcode concept [7], are also unsatisfactory for applying to asexual organisms because they require arbitrary cut-offs to determine the level at which a clade is considered a species. Whether there is a barcoding gap [8] or a coalescent point [9] is irrelevant for delimitation of asexual lineages, as neither have biological meaning. They simply represent patterns on phylogenies that could be the result of a plethora of causes, including extinction or under-sampling.

Some authors (e.g. [10]) have argued that the taxonomic rank we call “species” has no greater biological meaning than higher ranks such as “family” or “genus”. “Species” might simply be groups of individuals that are more closely related to one another than they are to other such groups, and this is probably more the case in asexual organisms than in obligatorily sexual ones. Nevertheless, species remain the fundamental units in systematic biology and ecology, and are essential for communication and application of conservation strategies [11] and quarantine decisions [12]. Confusion over species identity can have serious negative impacts on pest management programs [13] and international trade [14]. For these reasons, it is just as important to define “species” in asexual taxa as it is for sexual taxa, even though the biological meaning might differ.

Some studies dealing with species delimitation in asexual taxa, such as bdelloid rotifers (Rotifera: Bdelloidea: Philodinavidae) (e.g. [15–17]) and darwinulid ostracods (Crustacea: Ostracoda) (e.g. [18]), have defined species on an ecological basis, i.e., if two or more lineages have sorted into different niches, then each lineage may be considered to be a distinct species. The authors of these studies suggested that the main drivers of speciation are the same in sexual and asexual organisms (population isolation and adaptive selection), but the boundaries between asexual species are formed by processes other than sexual reproduction (an idea shared by De Queiroz [19]).

Here we investigate species delimitation in *Parasaissetia nigra* (Nietner, 1861) (Hemiptera: Sternorrhyncha: Coccoidea: Coccidae), the “nigra scale” or “black scale insect”, which is a polyphagous scale insect that feeds on plants from more than 80 families [20–21]. The species is thought to have originated in Africa [22] but is now widespread throughout the world. It is an important pest of ornamental and greenhouse plants [23], fruits and other plantations in tropical and subtropical countries [24–27], and is a moderate pest of several crops in temperate regions [22]. *Parasaissetia nigra* has also been identified as a potentially invasive species in areas of environmental concern, such as the Galápagos Islands [28]. Infestation by the insect can reduce the vigor of host plants by depleting their sap reserves, and the honeydew produced by the insects forms a suitable medium for the growth of sooty molds, which can lower the market value of fruits and ornamental plants [22].

It is generally accepted that females of *P*. *nigra* reproduce parthenogenetically [24, 29–31]: males have been reported twice [32–33], but there has been no evidence presented that show a direct connection between these specimens and *P*. *nigra*. Information that could confirm the existence of males in *P*. *nigra* (such as the existence of sperm bundles in the spermatheca or heterochromatic chromosomes in embryos, e.g. [34]) has not been documented in the species. If males do exist they must be rare: *P*. *nigra* is common, frequently collected and intensively studied worldwide, yet these two reports remain the only suggestion of the existence of males.

De Lotto [35–36] reported that there was considerable variation in the size, form and color of specimens of *P*. *nigra* from different areas of Africa, but could find no consistent morphological differences to warrant recognition of distinct species. Ben-Dov [30] examined specimens of *P*. *nigra* from across its range and reported differences in numbers of marginal setae, preopercular pores, submarginal tubercles and pocket-like sclerotisations, and different lengths of antennae and legs. Similarly, Hodgson [37] reported that adult females collected from Chad and Laos differed from those of other countries. Both Hodgson and Ben-Dov thought morphological differences might be linked to cryptic species diversity within *P*. *nigra*, but neither thought the evidence conclusive.

In this study, we aim to address the following questions:

1. Is *P*. *nigra* a single species with broad host-use and a cosmopolitan distribution, or should the clade be considered to represent more than one species?
2. If considered multiple species, are those species ecologically distinct, e.g., associated with specific host plants, geographic locations or climatic variables?

We use both single (*COI*) and multi-locus (*18S*, *28S*, *COI*, *Dynamin* and *EF-1α*) sequence data and multiple coalescent approaches [38–39] to determine the number of genetic clusters within *P*. *nigra* that could be recognized as species under coalescence-based species delimitation [40]. Alternative delineation hypotheses were tested under coalescent approaches and recent speciation events were reconstructed by applying probabilistic models. However, because there is no (or little) possibility of genetic exchange between individuals of a strictly (or largely) parthenogenetic organism, other than from mother to daughter, we need additional criteria for determining species boundaries that are testable and have some meaning for end users of the taxonomy.

Mallet [41] argues that conspecific individuals have common gene pools (*sensu* Dobzhansky [3–4]), and treats species as separately identifiable genotypic clusters. However, reproductive isolation is not viewed as the main mechanism stopping the exchange of genetic material among clusters. Under this concept, species boundaries are maintained by selection, and different species should have different ecological and/or geographic distributions—isolating mechanisms that Van Valen [42] and Andersson [43] used when applying the ecological species concept to delineate plants. We use a modification of this approach, defining species within parthenogenetic lineages as genetic clusters that are ecologically differentiated from other such clusters. The possibility of this resulting in paraphyletic species is discussed.

## Materials and methods

### Taxon sampling and DNA extraction

*Parasaissetia* Takahashi [44], of which *P*. *nigra* is the type, currently includes five species [20]. The first description of *P*. *nigra* was very brief [45] and included few of the taxonomic characters that are considered important today [30]. The redescriptions by Smith [29], De Lotto [36], Ben-Dov [30] and Hodgson [37] have allowed for an easier taxonomic interpretation. In the present study, 65 samples identified as *P*. *nigra* were included in molecular analyses. All were collected from outdoors and they represent populations from at least 50 different host plant species (25 families) and 44 localities across four continents and multiple islands (Table 1). The four other described species of *Parasaissetia* could not be included as specimens were not available for DNA extraction, but these are all morphologically different and clearly distinguishable from *P*. *nigra* [22]. Four species of *Saissetia* Déplanche, including the type species *S*. *coffeae* (Walker), were chosen as outgroups because the genus is closely related to *P*. *nigra* [46]. We included *C*. *longulus* (Douglas) because it is also closely related to *P*. *nigra* [47–48]. A specimen of *C*. *hesperidum* L. was used to root the trees because it represents a different but closely related tribe, Coccini [49].

**Table 1:** Samples of Coccidae used in this study.

Sample ID	*COI* clade	Host	Host family	Location	Geocode (WGS84)	Date of collection	Collector
**INGROUPS**							
***Parasaissetia nigra* (Nietner)**							
YPL00499	ANZ	*Pittosporum eugenioides*	Pittosporaceae	Napier, NZL	-38.488, 177.314	09.ii.2011	D. L. Brunt
YPL00500	ANZ	*Polygala* sp.	Polygalaceae	Auckland, NZL	-36.924, 174.836	15.ii.2011	C. Inghs
YPL00525	ANZ	*Melaleuca* sp.	Myrtaceae	Melbourne, VIC, AUS	-37.796, 144.964	06.xii.2011	Y.-P. Lin
YPL00544	ANZ	*Citrus* sp.	Rutaceae	Canberra, ACT, AUS	-35.256, 149.115	19.xii.2011	Y.-P. Lin
YPL00556	ANZ	*Macrozamia communis*	Zamiaceae	Murramarang Nat. Pk, NSW, AUS	-35.646, 150.283	13.i.2012	R. D. Edwards
YPL00573	ANZ	Unidentified plant	n.a.	Hobart, TAS, AUS	-42.849, 147.103	25.xi.2012	Y.-P. Lin
YPL00574	ANZ	*Polygala* sp.	Ploygalaceae	Eucla, WA, AUS	-31.680, 128.880	05.xi.2012	L. G. Cook
YPL00697	ANZ	*Melaleuca ericifolia*	Myrtaceae	Canberra, ACT, AUS	-35.164, 149.064	04.i.2014	B. Choi
YPL00238	G	unidentified tree	n.a.	Ankasa, GHA	5.813, -2.753	08.vi.2005	T. Kondo
YPL00239	G	unidentified tree	n.a.	Ankasa, GHA	5.813, -2.753	09.vi.2005	T. Kondo
YPL00243	ICB	*Pseudocedrela kotschingii*	Meliaceae	Zanzan, CIV	10.012, -12.012	ix.2001	B. Fiala
YPL00540	ICB	*Pseudocedrela kotschingii*	Meliaceae	Zanzan, CIV	10.012, -12.012	ix.2001	B. Fiala
YPL00734	ICB	*Ixora coccinea*	Rubiaceae	Calavi, BEN	6.419, 2.325	13.v.2015	G. Goergen
YPL00019	W1	*Alnus formosana*	Betulaceae	Chiayi City, TWN	23.472, 120.484	03.xi.2008	Y.-P. Lin
YPL00099	W1	*Musa supientum*	Musaceae	Chiayi County, TWN	23.454, 120.473	24.i.2009	Y.-P. Lin
YPL00361	W1	*Cordia dichotoma*	Boraginaceae	Chiayi County, TWN	23.442, 120.567	14.ii.2010	Y.-P. Lin
YPL00474	W1	*Annona montana*	Annonaceae	Tainan City, TWN	23.339, 120.503	16.i.2011	Y.-P. Lin
YPL00477	W1	*Bischofia javanica*	Euphorbiaceae	Tainan City, TWN	23.339, 120.503	16.i.2011	Y.-P. Lin
YPL00478	W1	*Melastoma candidum*	Melastomataceae	Tainan City, TWN	23.339, 120.503	16.i.2011	Y.-P. Lin
YPL00548	W1	*Gardenia* sp.	Rubiaceae	Tainan City, TWN	23.187, 120.485	19.i.2012	Y.-P. Lin
YPL00551	W1	*Cordia dichotoma*	Boraginaceae	Tainan City, TWN	23.180, 120.566	19.i.2012	Y.-P. Lin
YPL00723	W1	*Trichospermum pleiostigma*	Malvaceae	Ramu River Basin, Madang, PNG	-5.140, 145.110	07.ix.2007	P. Klimes
YPL00011	W2	*Tabebuia chrysantha*	Bignoniaceae	Chiayi City, TWN	23.491, 120.449	30.x.2008	Y.-P. Lin
YPL00118	W2	*Ficus microcarpa*	Moraceae	Kinmen, TWN	24.457, 118.345	03.ii.2009	Y.-P. Lin
YPL00119	W2	*Macaranga tanarius*	Euphorbiaceae	New Taipei City, TWN	25.154, 121.459	06.ii.2009	Y.-P. Lin
YPL00126	W2	*Zelkova serrata*	Ulmaceae	Yilan County, TWN	24.683, 121.754	08.ii.2009	Y.-P. Lin
YPL00337	W2	*Psidium guajava*	Myrtaceae	Chiayi City, TWN	23.496, 120.432	15.xii.2009	Y.-P. Lin
YPL00340	W2	*Ehretia microphylla*	Boraginaceae	Chiayi County, TWN	23.537, 120.351	22.xii.2009	Y.-P. Lin
YPL00364	W2	*Tetrapanax papyriferus*	Araliaceae	Hualien County, TWN	23.985, 121.612	21.ii.2010	Y.-P. Lin
YPL00426	W2	*Cordia dichotoma*	Boraginaceae	Taitung County, TWN	22.771, 121.146	29.iv.2010	Y.-P. Lin
YPL00473	W2	*Ehretia resinosa*	Boraginaceae	Kaohsiung City, TWN	22.681, 120.301	22.xii.2010	Y.-P. Lin
YPL00476	W2	*Ficus irisana*	Moraceae	Tainan City, TWN	23.339, 120.503	16.i.2011	Y.-P. Lin
YPL00483	W2	*Croton tiglium*	Euphorbiaceae	Taitung County, TWN	22.687, 120.991	20.i.2011	Y.-P. Lin
YPL00487	W2	*Ficus* sp.	Moraceae	Taitung County, TWN	22.691, 120.999	20.i.2011	Y.-P. Lin
YPL00492	W2	*Plumeria obtusa*	Apocynaceae	Heshan, Guangtung, CHN	22.472, 112.732	27.i.2011	Y.-P. Lin
YPL00495	W2	*Psidium guajava*	Myrtaceae	Heshan, Guangtung, CHN	22.472, 112.732	27.i.2011	Y.-P. Lin
YPL00562	W2	Unidentified plant	n.a.	North Keeling, CCK	-10.384, 94.056	26.iii.2012	G. Neumann
YPL00620	W2	*Manihot esculenta*	Euphorbiaceae	Milingimbi, NT, AUS	-12.660, 134.554	22.vi.2011	L. Halling
YPL00699	W2	*Ficus* sp.	Moraceae	Naha, Okinawa, JPN	26.211, 127.688	19.xii.2014	Y.-P. Lin
YPL00073	W3	*Morus* sp.	Moraceae	Brisbane, QLD, AUS	-27.494, 153.015	17.xi.2008	Y.-P. Lin
YPL00075	W3	*Xanthostemon chrysanthos*	Myrtaceae	Brisbane, QLD, AUS	-27.476, 153.039	20.xi.2008	Y.-P. Lin
YPL00078	W3	*Harpullia pendula*	Sapindaceae	Brisbane, QLD, AUS	-27.476, 153.039	27.xi.2008	Y.-P. Lin
YPL00080	W3	*Syzygium australe*	Myrtaceae	Brisbane, QLD, AUS	-27.460, 152.976	27.xi.2008	L. G. Cook
YPL00083	W3	*Dodonaea* sp.	Sapindaceae	South West Rocks, NSW, AUS	-30.543, 153.024	28.xii.2008	L. G. Cook
YPL00085	W3	*Plumeria obtusa*	Apocynaceae	Brisbane, QLD, AUS	-27.500, 153.009	10.i.2009	Y.-P. Lin
YPL00089	W3	*Schinus terebinthifolius*	Anacardiaceae	Brisbane, QLD, AUS	-27.499, 153.012	10.i.2009	Y.-P. Lin
YPL00256	W3	*Buckinghamia celsissima*	Proteaceae	Brisbane, QLD, AUS	-27.498, 153.012	17.iv.2009	Y.-P. Lin
YPL00260	W3	*Macaranga* sp.	Euphorbiaceae	Brisbane, QLD, AUS	-27.476, 153.039	14.vi.2009	Y.-P. Lin
YPL00284	W3	*Syzygium* sp.	Myrtaceae	Goondiwindi, QLD, AUS	-28.418, 150.355	05.vii.2009	Y.-P. Lin
YPL00287	W3	unidentified tree	n.a.	North Stradbroke Island, QLD, AUS	-27.433, 153.521	07.viii.2009	Y.-P. Lin
YPL00289	W3	*Dodonaea triquetra*	Sapindaceae	North Stradbroke Island, QLD, AUS	-27.601,153.425	07.viii.2009	Y.-P. Lin
YPL00315	W3	*Monstera deliciosa*	Araceae	Brisbane, QLD, AUS	-27.501, 153.011	10.x.2009	Y.-P. Lin
YPL00323	W3	*Ravenala madagascariensis*	Strelitziaceae	Brisbane, QLD, AUS	-27.495, 153.014	02.xi.2009	Y.-P. Lin
YPL00356	W3	*Hymenosporum flavum*	Pittosporaceae	Brisbane, QLD, AUS	-27.498, 153.011	28.i.2010	M. Herne
YPL00449	W3	*Syzygium* sp.	Myrtaceae	Narrabari, NSW, AUS	-30.226, 149.777	12.xi.2010	Y.-P. Lin
YPL00462	W3	*Syzygium* sp.	Myrtaceae	Kuala Lumpur, MYS	3.143, 101.688	13.xii.2010	Y.-P. Lin
YPL00498	W3	*Dodonaea* sp.	Sapindaceae	South West Rocks, NSW, AUS	-30.971, 152.834	01.i.2011	L. G. Cook
YPL00522	W3	*Psidium guajava*	Myrtaceae	North Stradbroke Island, QLD, AUS	-27.528, 153.284	20.xi.2011	Y.-P. Lin
YPL00578	W3	*Philodendron* sp.	Araceae	Knockrow, NSW, AUS	-28.761, 153.540	03.xi.2013	Y.-P. Lin
YPL00688	W3	*Cupaniopsis anacardioides*	Sapindaceae	Myall Shores, NSW, AUS	-32.519, 152.223	03.xi.2014	Y.-P. Lin
YPL00692	W3	*Hymenosporum* sp.	Pittosporaceae	Adelaide, SA, AUS	-34.901, 138.571	16.xi.2014	Y.-P. Lin
TK0151	W3	Unidentified plant	n.a.	Phisanulok, THA	16.707, 98.048	2002	P. Cranston
TK0177	W3	*Monstera* sp.	Araceae	Santa Rosa de Cabal, Risaralda, COL	6.332, -78.226	08.i.2005	T. Kondo
TK0187	W3	Unidentified plant	Arecaceae	Pance (near Cali), Valle, COL	6.385, -84.493	28.xii.2005	T. Kondo
TK0205	W3	*Hedera* sp.	Araliaceae	Halfway House, Gauteng, ZAF	-28.966, 27.273	30.v.2005	I. Millar
**OUTGROUPS**							
***Coccus hesperidum* Linnaeus**							
YPL00076		*Morus* sp.	Moraceae	Brisbane, QLD, AUS		20.xi.2008	Y.-P. Lin
***C*. *longulus* (Douglas)**							
YPL00433		*Acacia* sp.	Fabaceae	Sunshine Coast, QLD, AUS		13.vi.2010	Y.-P. Lin
***Saissetia coffeae* (Walker)**							
YPL00104		*Cordia dichotoma*	Boraginaceae	Chiayi County, TWN		26.i.2009	Y.-P. Lin
***S*. *miranda* (Cockerell & Parrott in Cockerell)**							
YPL00032		*Mangifera indica*	Anacardiaceae	Chiayi County, TWN		05.xi.2008	Y.-P. Lin
***S*. *oleae* (Olivier)**							
YPL00246		*Heteromeles arbutifolia*	Rosaceae	Davis, CA, USA		01.iv.2009	Y.-P. Lin
***S*. *somereni* (Newstead)**							
YPL00237		*Theobroma cacao*	Malvaceae	Ankasa, GHA		08.vi.2005	T. Kondo

Abbreviations: ACT: Australian Capital Territory; AUS: Australia; BEN: Benin; CA: California; CCK: Cocos Islands; CHN: China; COL: Colombia; GHA: Ghana; CIV: Côte d'Ivoire; JPN; Japan; MYS: Malaysia; NSW: New South Wales; NT: Northern Territory; NZL: New Zealand; PNG: Papua New Guinea; QLD: Queensland; SA: South Australia; TAS: Tasmania; THA: Thailand; TWN: Taiwan; USA: United States; VIC: Victoria; WA: Western Australia; ZAF: South Africa. The COI clade to which each specimen of Parasaissetia nigra belongs is indicated in the second column. The map data were based on Geocode (WGS84).

Insects collected in the field were preserved in absolute ethanol (> 99.5%; p.a.) and then stored at 4°C. Genomic DNA was extracted from young adult females or post-reproductive females containing eggs and crawlers (first instar nymphs) using a CTAB/chloroform protocol or a DNeasy Blood & Tissue kit (cat. no. 69504, Qiagen, Hilden, Germany) as per Lin *et al*. [47]. After DNA extraction, cuticles were slide-mounted as vouchers using the method of Ben-Dov & Hodgson [50]. All slides will be deposited in the Australian National Insect Collection, Canberra, Australia. Specimens of *P*. *nigra* were identified following the detailed descriptions of adult females by Ben-Dov [30] and Hodgson [37]. The identification of outgroup species was based on De Lotto [35] (*S*. *somereni*), Williams & Watson [51] (*C*. *longulus*, *S*. *miranda* and *S*. *oleae*) and Hodgson [37] (*C*. *hesperidum* and *S*. *coffeae*).

### PCR, clean-up and gel purification

Five genes from four independent loci (including mitochondrial and nuclear regions) were amplified: the nuclear ribosomal regions *18S* SSU (5’ region) and *28S* LSU (D2 and D3 regions), which occur as numerous tandem repeats [52], the mitochondrial gene *COI* [53], and the nuclear protein-coding genes with low- (*EF-1α*) [54] or single- (*Dynamin*) [55] copy number in other insects.

We used the same primer pairs and PCR programs for amplifying *18S*, *28S*, *Dynamin* and *EF-1α* as Lin *et al*. [47] (Table 2). Three primer pairs were used for amplifying two regions of *COI* (Table 2). CI-J-2183 (Jerry) and C1-N-2568 (Ben) (Table 2) was used for a 3' region of *COI* with a step-down PCR program (Table 2). The barcode region of *COI* was amplified with primer pair PcoF1 and HCO for the six outgroup taxa but, because this combination did not work well for *P*. *nigra*, HCO was replaced with newly designed primer (nigra_Ben) (Table 2) modified from C1-N-2568. A negative control was used for all PCR reactions and each 25 μL PCR mixture comprised 5 μL 5x PCR buffer, 2 μL dNTP (2mM), 1.5 μL MgCl_2_ (50 mM), 0.5 μL of each forward and reverse primer (10 μM), 0.15 μL (1.5 U) Taq-polymerase (MangoTaq, cat. no. BIO-21083, Bioline, Australia), 2 μL (*18S*, *28S* and *COI* reactions) or 4 μL (*Dynamin* and *EF-1α* reactions) of template, and 13.35 μL or 11.35 μL ddH_2_O (UltraPure^™^ DNAse/RNAse-Free Distilled Water, cat. no. 10977, Invitrogen, Australia).

**Table 2:** Primers and PCR protocols used.

Gene region	Primer	Direction	Primer sequence 5’ to 3’	Annealing temperature	Reference
*28S* D2/D3	S3660	F	GAGAGTTMAASAGTACGTGAAAC	55°C	[56]
	A335	R	TCGGARGGAACCAGCTACTA		[57]
*18S*	2880	F	CTGGTTGATCCTGCCAGTAG	55°C	[58]
	B-	R	CCGCGGCTGCTGGCACCAGA		[58]
*COI*	PcoF1	F	CCTTCAACTAATCATAAAAATATYAG	45°C/51°C	[59]
	HCO	R	TAAACTTCAGGGTGACCAAAAAATCA		[60]
	nigra_Ben	R	GCRATTACATAATATGTATCATG		This study
	CI-J-2183 (Jerry)	F	CAACATTTATTTTGATTTTTTGG	Step-down to 42°C*	[53]
	C1-N-2568 (Ben)	R	GCWACWACRTAATAKGTATCATG		[61]
*Dynamin*	3006F1.1	F	CCGGAYATGGCGTTCGAAGCTA	50°C	[55]
	3006R2.1	R	TCTTCGTGGTTGGTGTTCATGTACGC		[55]
*EF-1α*	scutA_F	F	ATTGTCGCTGCTGGTACCGGTGAATT	50°C	[62]
	rcM52.6	R	GCYTCGTGGTGCATYTCSAC		[54]

* The program had a 4 min denaturation at 94°C, an original annealing temperature of 65°C and a 45 s extension at 72°C. For each additional cycle the annealing temperature was reduced by 5°C until reaching 42°C. An additional 30 cycles with an annealing temperature of 42°C was subsequently run. The final extension was at 72°C for 3 min.

PCR products were cleaned using Exonuclease I and Antarctic Phosphatase (cat. no. M0293S and M0289S, New England BioLabs, Australia), or with the ammonium acetate/ethanol precipitation method used by Lin *et al*. [47]. In amplicons with multiple bands, the target band was excised from a 1% agarose gel under UV illumination and purified using the Wizard SV Gel and PCR Clean-up System (cat. no. #A9281, Promega, Madison, USA) following the manufacturer’s instructions. All PCR products were sequenced using Sanger sequencing at Macrogen Inc. (Republic of Korea).

### Sequence editing and alignment

Sequences were edited using MEGA5 [63], then imported and aligned visually in Se-Al v.2.0 [64]. Translations to amino acids were used to assist alignment of the three protein coding regions (*COI*, *Dynamin* and *EF-1α*) and to check for stop codons. Intron-exon boundaries of *Dynamin* and *EF-1α* were assigned using the GT-AG rule [65] and comparison with annotated sequences in GenBank.

### Identification of clades/lineages

We used phylogenetic methods to identify clades common to individual gene trees and analyses of concatenated data that could represent putative species, and thus form the basis for additional analyses. Most methods of phylogeny estimation assume that base composition among taxa is homogeneous [66] so we firstly checked for base frequency bias among taxa (non-stationarity) in all datasets using PAUP* 4.0b10 [67]. Each codon position for the three protein-coding regions was tested independently, with and without invariant sites.

We used Bayesian inference (BI) implemented in MrBayes v.3.2.1 [68] to analyze a concatenated dataset and each gene region separately, except that we grouped the nrDNA loci *18S* and *28S* given that they are physically linked in the ribosomal array. Outgroups were used to root the phylogenies and introns were excluded because they could not be unambiguously aligned across the distantly related taxa.

Substitution models for each partition were selected using MrModeltest 2.3 [69] (Table 3). The *COI*, *Dynamin* and *EF-1α* datasets were each assigned two partitions: first plus second codon positions, and third codon positions. The GTR [70] + I + G model (nst = 6, rates = invgamma) was chosen for each partition of *COI*, *EF-1α* and *28S*. A K2P [71] + I + G model was applied to *18S*, and a JC69 [72] model and K2P + I + G model was selected for the two partitions of *Dynamin*. Each analysis comprised two independent runs of 90 million (nrDNA), 60 million (*COI*, *Dynamin* and *EF-1α*) or 40 million (concatenated dataset) generations with the default setting of four Markov chains (three heated and one cold), temperature = 0.10, starting from a random tree and sampling every 1000th generation.

**Table 3:** DNA substitution models, the length of runs and the number of burn-in used for each gene region in BI analyses.

Gene	Model	Runs (million generations)	Burn-in (million generations)
nrDNA (*18S*+*28S*)	GTR+I+G (*28S*), K2P+I+G (*18S*)	90	60
*COI*	GTR+I+G	60	50
*Dynamin*	JC69 (first partition), K2P+I+G (second partition)	60	24
*EF-1α*	GTR+I+G	60	20
Concatenated		40	30

The performance of each Bayesian analysis was checked by examining the average standard deviation of split frequencies (should be less than 0.01) [73], PSRF values (should be close to 1.00) [68], the absolute value of the difference between the harmonic means of the two runs (should be less than 2) [74] and the ESS (Effective Sample Size, should be > 200) [75] of each statistic determined using Tracer v.1.6 [76]. The number of trees discarded as the burn-in period varied with each analysis, depending on when stationarity was reached (60 (*18S* + *28S*), 50 (*COI*), 24 (*Dynamin*), 20 (*EF-1α*) and 30 (concatenated) million generations respectively.). A maximum clade credibility (MCC) tree with posterior probability values from the two runs of each analysis was selected using TreeAnnotator [77] and the post-burn-in trees.

As another check of clade membership, we also analyzed each dataset using maximum parsimony (MP) using PAUP* 4.0b10 [67] (S1 Appendix) because it has different underlying assumptions from BI and congruence among them indicates results are not sensitive to the method of phylogeny estimation.

Because introns and hypervariable regions of rDNA had to be excluded in the outgroup-rooted analyses, we used BEAST 1.8.0 [77] to analyze a concatenated dataset that included only sequences from *P*. *nigra* but which included these regions. Outgroups are unnecessary in BEAST because this software roots trees by applying a (calibrated or implicit) molecular clock [75].

XML files for BEAST were generated using BEAUTI 1.8.0. [77]. Partitioning was the same as for the MrBayes analyses except for the addition of intron partitions for *Dynamin* and *EF-1α*. A HKY [78] + I + G was used for all partitions except the exons of *Dynamin*, for which a K2P + I model was applied.

A gamma distribution with initial value, shape and scale set to 1.0, 0.001 and 1000 was used as the prior for these analyses. The exon and intron regions of *Dynamin* and *EF-1α* were treated separately and each had the same, but unlinked, clock model. The tree prior was set to the pure birth Yule speciation process [79], which assumes that all lineages speciate at the same rate in any given time period and have uniform birth rates (prior distribution low = 0.0; upper = 1.0E100; initial = 1.0). This is the simplest branching model in species-level processes [80].

Each run comprised 50 million generations starting from a random tree and was sampled every 1000th generation. The performance of each BEAST run was checked by examining the ESS values of each statistic shown by Tracer v.1.6. A maximum clade credibility tree was chosen from the posterior set using TreeAnnotator and after removing samples from the burn-in period.

### Multi-locus coalescent species delimitation (*BEAST)

Using the same partitioning schemes and models as those applied for BEAST, we tested multiple species hypotheses based on supported clades recovered in analyses of individual and concatenated datasets that had a *COI* divergence >2%. Eight species hypotheses, ranging from two to six species, were tested (Table 4) by assigning terminals to different taxa and comparing the marginal likelihood values of the different hypotheses after the species tree was generated.

**Table 4:** The eight different species hypotheses tested using *BEAST.

Hypothesis	Species (clades) tested	Supporting dataset
2Sp	(ANZ + W1), (IC + G + W2 + W3)	Con, *COI*, *Dynamin*, *EF1a*
3Sp1	ANZ, W1, (IC + G + W2 + W3)	Con, *COI*, *Dynamin*, *EF1a*
3Sp2	(ANZ + W1), IC, (G + W2 + W3)	Con, *COI*
4Sp	ANZ, W1, IC, (G + W2 + W3)	Con, *COI*
5Sp1	ANZ, W1, IC, (G +W2), W3	Con, *COI*
5Sp2	ANZ, W1, IC, G, (W2 + W3)	Con, *COI*
5Sp3	ANZ, W1, IC, W2, (G + W3)	Con, *COI*
6Sp	ANZ, W1, IC, G, W2, W3	Con, *COI*, *Dynamin*

**Fig 1:**
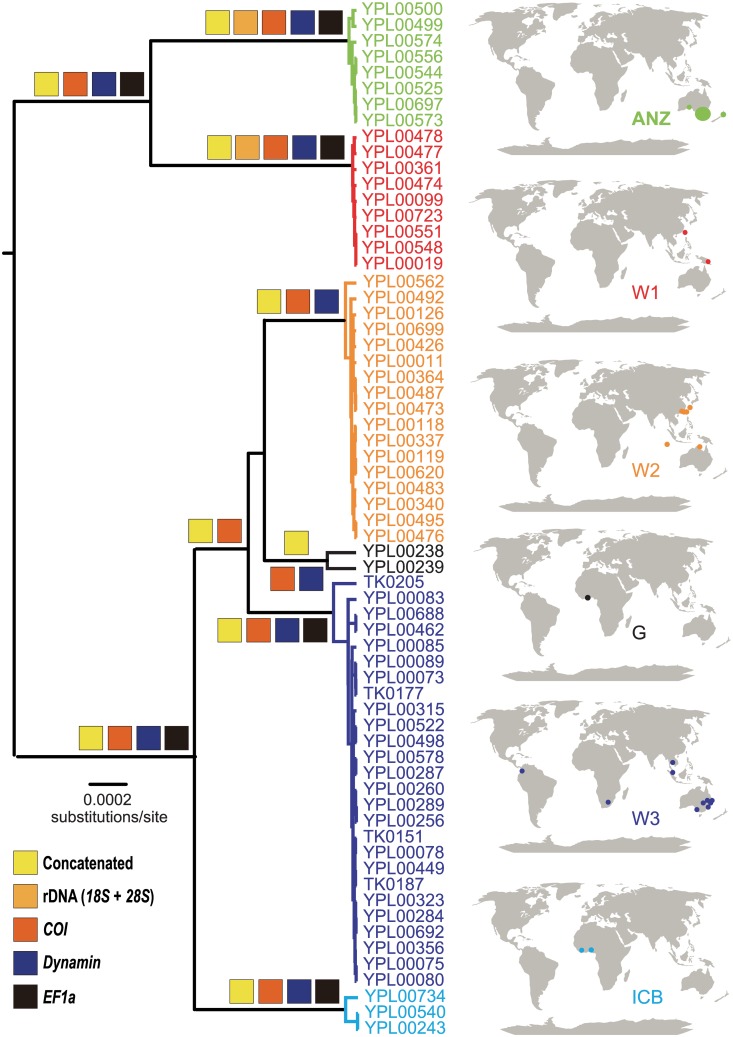
BEAST maximum clade credibility (MCC) tree inferred using concatenated dataset (3454 bp) and 65 specimens of *Parasaissetia nigra*. Specimens are color-coded by clade and their collection localities are indicated by the same color in the adjacent maps. The colored squares above branches indicate that the branch was strongly supported (Bayesian posterior probability ≥ 0.95) in analyses of that dataset. Branch supports within each clade are not shown.

Con: concatenated dataset. Clade names are based on Fig 1.

Each run started from a random tree and was sampled every 5000 generations. We implemented two models of population size, “piecewise linear and constant root” (PLCR) and “piecewise linear” (PL), rather than the piecewise constant population size model because *P*. *nigra* is extremely widespread [20] and is likely to have undergone a population expansion. Each hypothesis was run under both Yule [79] and birth-death [81] speciation processes separately. Convergence of each *BEAST run was assessed in the same manner as for BEAST. The number of generations/run (200 to 600 million) and the burn-in (5 to 200 million) required for stationarity varied for each analysis.

The harmonic means [82] of runs for each species hypothesis were estimated in BEAST 1.8.0. The favored hypothesis was that with the lowest harmonic mean that was significantly different from other means, with significant difference assessed using Bayes Factors [74]. Because the harmonic mean method might not be sufficiently sensitive [83], we also used a posterior simulation-based approach (Akaike’s information criterion through Markov chain Monte Carlo (AICM) comparisons [84]) to identify the best species hypothesis. The hypothesis with the lowest AICM was assumed to be best, with the *P* values from calculating the exponential value ((minimum AICM–maximum AICM)/2) indicating whether any two hypotheses were significantly different from each other (*P* < 0.05, d.f. = 1).

### Single-locus coalescent species delimitation (GMYC)

We used the single-threshold General Mixed Yule Coalescent (GMYC) method [85] on the *COI* sequences of *P*. *nigra*. GMYC estimates the number of clusters (“species”) by recognizing the transitions from between- to within-species branching patterns on an ultrametric and fully dichotomous tree. A likelihood ratio (LR) test was applied to determine whether the mixed coalescent and Yule stochastic lineage growth (GMYC) model provided a better fit to the data than the null model, which hypothesizes that the entire sample belongs to a single species with no shift in branching processes. The ultrametric tree needed as input was estimated using BEAST with a strict clock, the substitution rate set to 1.0 (as suggested by Gamble *et al*. [86]), and two partitions (first + second codon positions: third codon position) with an HKY+I+G substitution model for each. The GMYC analysis was implemented using the APE [87] and SPLITS [88] packages in R [89].

### Environmental niche modelling

To determine whether there is evidence of ecological differentiation among the identified lineages within *P*. *nigra*, which could be interpreted to indicate the presence of distinct ecological species, we compared the geographic distribution and host-use of each clade.

Poor representation of most lineages precluded a thorough assessment of geographical and ecological limits to distribution, but ANZ (from 6 localities) and W3 (23 localities) lineages have wide distributions and relatively good sampling from Australia. This allowed a comparison of the lineages across a continental-scale rainfall and temperature gradient. The two collecting localities of *P*. *nigra* (W3 lineage) from Rakimov *et al*. [48] were included in our species distribution modelling to increase sample size. We used the geocode for each collection and initially modelled the distribution of each lineage in MaxEnt v. 3.3.3k [90] using the "Bioclim" dataset [91] of 19 high resolution (c. 1 km at the equator) layers (S2 Appendix). We then used the six top performing variables (those contributing ≥ 10% to the model) (S2 Appendix) in an identity test run using ENMTools (perl script version 1.0) [92]. We limited the area considered to a background to the geographic extent determined in the original full Bioclim MaxEnt model: therefore, the question addressed by the identity test was "In eastern and southern Australia, do ANZ and W3 occupy environments that are less similar than expected by chance?".

## Results

Sequence data for all five DNA regions were obtained for all specimens except that *Dynamin* could not be amplified for three (S3 Appendix). GenBank accession numbers of sequences are given in S3 Appendix. No base composition bias (non-stationarity) was detected among taxa in any of the datasets (*P* = 1.00 in all). Stationarity and convergence between runs was reached in all Bayesian analyses.

### Identification of lineages/clades

There was strong support for the sampled specimens of *P*. *nigra* forming a clade in all analyses using outgroup-rooting (bootstrap values (BS) ≥ 70% in maximum parsimony and Bayesian posterior probabilities (PP) = 1.00) (S1 Fig). Additionally, analyses of *COI* (S2 Fig), *Dynamin* (S3 Fig) and concatenated datasets (Fig 1) in BEAST recovered six congruent clades within *P*. *nigra* that each had strong support (PP = 0.99 to 1.00 for BI). We named each on the basis of geographic distribution: Australia and New Zealand (ANZ), Côte d'Ivoire and Benin (ICB), Ghana (G), and widespread (W): Papua New Guinea and Taiwan (W1), Northern Territory (Australia), China, North Keeling, Okinawa and Taiwan (W2), and Australia, Colombia, Malaysia, South Africa and Thailand (W3) (Fig 1). The *COI* uncorrected genetic distances (p-distances) within the six clades ranged from 0.0–0.9%, and between the clades ranged from 3.0–10.0% (Table 5).

**Table 5:** The % pair-wise distances (uncorrected) in *COI* between and within clades of *Parasaissetia nigra*.

	**ANZ**	**W1**	**ICB**	**G**	**W2**	**W3**
**ANZ**	0.0					
**W1**	7.2	0.0				
**ICB**	8.8–8.9	10.0–10.1	0.0–0.4			
**G**	9.1–9.4	9.1–9.2	6.6–6.9	0.9		
**W2**	9.0–9.2	9.3–9.4	6.5–6.8	3.1–3.8	0.0–0.2	
**W3**	8.7–8.9	9.1–9.4	5.5–6.1	3.0–3.8	3.0–3.3	0.0–0.5

Five of the clades (ANZ, W1, ICB, W2 and W3) were also strongly supported (PP > 0.99) in the chronogram of *EF-1α* (S4 Fig), but the two samples from Ghana (G) were not monophyletic. Only ANZ, W1 and W2 were recovered in analyses of *18S* + *28S* (S5 Fig), with strong support only for ANZ and W1 (PP = 0.97 and 1.00 respectively).

### Tests of species hypotheses using *BEAST

Because clades ANZ and W1 were well supported in the BEAST-generated chronograms of all datasets, the two clades were treated as distinct species except in two hypotheses (Sp2 and 3Sp2) that combined the two clades into a single species (Table 4). Because sequences of the two specimens from Ghana (YPL00238 and YPL00239) did not cluster together in BEAST analyses of *EF-1α* (S4 Fig) or rDNA (S5 Fig), five alternative scenarios for their relationships were developed (3Sp1, 4Sp, 5Sp1, 5Sp2 and 5Sp3) (Table 4). Finally, the 6Sp he hypothesis treated the six lineages as different species.

Except for hypothesis 5Sp3, which had low ESS (< 50) in all priors linked to *EF-1α* even after 600 million generations, all *BEAST runs reached stationarity. With the exception of the 2Sp, 3Sp2 and 5Sp2 hypotheses, there were no significant differences between results using the PLCR and PL population models under both the Yule and Birth-death speciation processes, as assessed by AICM (*P* < 0.05) and harmonic means (absolute value of difference > 6) (Table 6). Both Bayes Factors and AICM indicated that the 6Sp hypothesis was the best, regardless of speciation and population size models (Table 6), but this model was only significantly better than the second best hypothesis (5Sp2) in the AICM from the combination of Birth-Death and PL models (Table 6).

**Table 6:** The results for the seven species hypotheses that reached convergence in *BEAST analyses.

Hypothesis	Population size model	Yule			Birth-Death		
		Generations/Burn-in	HM	AICM	Generations/Burn-in	HM	AICM
2Sp	PLCR	500M/50M	-6400.77	12966.58	500M/50M	-6407.51	12968.59
	PL	500M/50M	-6404.17	12967.55	500M/50M	-6407.86	12961.87
3Sp1	PLCR	600M/6M	-6416.60	12995.01	600M/6M	-6414.64	12992.70
	PL	200M/20M	-6412.56	12992.46	500M/80M	-6416.75	12998.64
3Sp2	PLCR	200M/75M	-6389.02	12914.46	200M/75M	-6391.61	12905.25
	PL	200M/75M	-6392.33	12922.92	200M/20M	-6388.23	12903.29
4Sp	PLCR	500M/50M	-6396.34	12918.70	500M/50M	-6396.79	12922.02
	PL	500M/50M	-6399.60	12923.15	500M/200M	-6394.21	12921.37
5Sp1	PLCR	500M/50M	-6400.81	12912.97	500M/5M	-6400.30	12912.20
	PL	500M/50M	-6401.67	12913.86	500M/5M	-6403.78	12913.59
5Sp2	PLCR	400M/40M	-6388.67	12881.97	500M/20M	-6391.02	12885.41
	PL	500M/5M	-6395.76	12887.25	500M/10M	-6395.50	12890.54
6Sp	PLCR	500M/50M	-6392.84	12882.68	500M/50M	-6393.47	12880.26
	PL	300M/30M	-6393.39	12886.23	500M/50M	-6393.03	12879.05

All 65 specimens of *Parasaissetia nigra* were included. PLCR = piecewise linear and constant root model, PL = piecewise linear model, HM = harmonic mean, AICM = Akaike’s information criterion through Markov chain Monte Carlo.

### Species delimitation using GMYC

Six ML entities ("species") (confidence interval: 6–6) (S6 Fig) were recognized after running the single threshold GMYC model (likelihood ratio = 31.03, *P* < 0.01). These six ML entities correspond to the six major clades found in the BEAST analyses of *COI*, *Dynamin* and concatenated datasets (Fig 1; S2 and S3 Figs), which also formed the basis of the 6Sp hypothesis in *BEAST analyses.

### Ecological differentiation

None of the lineages of *P*. *nigra* were clearly host specific and some hosts were shared across lineages (Table 1). For example, *Cordia dichotoma* was host to YPL00361 (clade W1) and YPL00426 (clade W2), and *Plumeria obtusa* (YPL00492 and YPL00085) and *Psidium guajava* (YPL00495 and YPL00522) were host to some specimens from clades W2 and W3.

**Fig 2:**
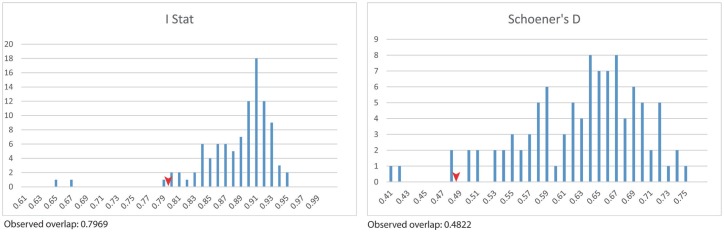
Identity test results using top 6 environmental layers cut down to model extent. Null distributions (100 replicates) are represented as blue bars with observed niche overlap between the ANZ and W3 clades indicated by the red arrows.

**Fig 3:**
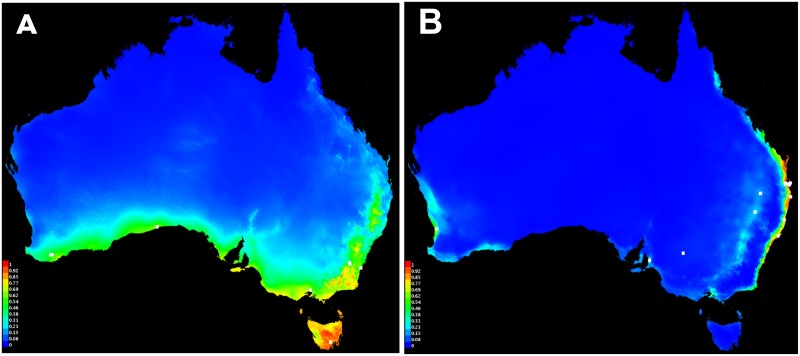
Species distribution models for the ANZ (A) and W3 (B) clades of *Parasaissetia nigra* in Australia, estimated using Maxent model and the 19 bioclim environmental layers. Collection localities used in the model are indicated by the white dots. Colder and warmer colors indicate the predictions of lower and higher probability of occurrence respectively.

Clades ANZ and W3 occupy different climatic zones in Australia (Identity test: Shoener's D and I statistic, 0.03 < P ≤ 0.04) (Fig 2). Temperature in the warmest period (bio 5) was the most important variable in the model for ANZ, whereas precipitation coldest quarter, precipitation warmest quarter, and annual mean temperature (bio19, 18 and 1 respectively) were most important for the model for W3. In general, ANZ is distributed in temperate areas (Fig 3A), whereas W3 is mostly restricted to coastal subtropical (eastern areas) and Mediterranean (regions around Adelaide and Perth) areas in Australia (Fig 3B).

## Discussion

Overall, we found at six deeply divergent lineages (>3% *COI* divergence; defined in Fig 1) within *P*. *nigra* that do not represent host-specific or geographically distinct clades. In sexual organisms, these divergent lineages could be considered distinct species under biological and evolutionary genetic species concepts. Given that they are parthenogenetic, if all lineages within *P*. *nigra* were equivalent in biology and ecology, recognizing only a single species could be justified given there is also no clear morphological differentiation. Here, however, we find that two lineages occurring in Australia and elsewhere appear to be ecologically distinct, occupying climatically different regions. We argue that ecological differentiation warrants the recognition of two species among these parthenogenetic lineages (ANZ and the rest). Below we discuss the practicalities of this, such as needing to recognize paraphyletic species—those lineages that have the same morphology and ecology but which do not form a monophyletic group.

### Species delimitation for biosecurity

How species are delimited can affect biosecurity, particularly in the areas of international and intra-national quarantine and trade. For example, products infested with invasive alien species are prohibited entry to some countries—the reason agricultural and other goods are inspected at state and national borders (e.g. [12]). If an invasive pest species is known to already occur in a region, products carrying that species might be allowed, whereas they might be prohibited or destroyed if the pest is not known to already occur there. Taxonomic "over-splitting" could be an unnecessary hindrance to trade, whereas "under-splitting" or "over-lumping" might lead to greater spread of invasive alien species and pose a threat to global agriculture. For asexual lineages, the delimitation of species is far more arbitrary than it is for obligatorily sexual ones—a criterion of no or little gene flow cannot be applied since every individual would be recognized as a species, and each species would have a short life span (several months in the case of *P*. *nigra*).

Application of so-called "objective" methods (e.g. [93]), such as multi-locus coalescence, has been proposed for use in delimiting asexual lineages (e.g. 4x rule, [16]). Coalescence is strongly influenced by the effective population size (*N*e), time, mutation rate and population structure of the target organisms (e.g. [94]) such that, if populations are large and exchange genes (migrants), coalescence will be deep. In coalescence-based methods of species delimitation using multi-locus data on sexual species, the number of species can be overestimated because population structure, rather than species boundaries, might be recovered [95]. Also, uneven sampling from across a species' range, such as we have for *P*. *nigra*, can also lead to coalescence that represents population structuring other than species boundaries (as in many barcoding studies reviewed by Lohse [96]).

Time to coalescence increases dramatically with reduction of gene flow or with increasing number of populations [97], and therefore coalescence is expected to be much deeper for asexual lineages (which have no gene flow and all individuals are "populations"). This means that depth of coalescence in asexual lineages is much deeper than that in sexual species, even when the number of individuals is the same. There is no "population structure" in asexual lineages, in the sense of differential likelihood of genes being exchanged, and no "speciation" in the sense of genetic isolation as a consequence of cessation of gene flow, thus coalescence is largely driven by mutation rate, *Ne* and extinction. Consequently, we argue that coalescence-based species delimitation methods, despite their occasional use for asexual organisms (e.g. [17, 98]), have little useful meaning for delimiting parthenogenetic species for biosecurity other than to apply the same method of delimitation as those applied to sexual species. Indeed, given that phylogeny alone provides an explanation for the pattern of traits in parthenogenetic organisms, any division of parthenogenetic lineages into species is arbitrary [99].

For parthenogenetic organisms of potential biosecurity concern, we suggest that the priority should be to identify ecologically distinct lineages as species, while minimizing any arbitrary species cut-offs, i.e. walking a tightrope between minimizing potential environmental or agricultural harm and minimizing potential disruption to trade. Morphological distinctions might sometimes be considered surrogates for ecological differences.

### Is *P*. *nigra* a cryptic species complex?

The current concept of *P*. *nigra* is that of a phenotypic cluster (*sensu* Sokal & Crovello [100])—a group of individuals that are morphologically similar to each other but phenotypically different from all other scale insects. If *P*. *nigra* was largely sexual, reciprocal monophyletic across mitochondrial and multiple nuclear genes (Fig 1; S2–S5 Figs), combined with the coalescence results found here (GYMC and *BEAST), would indicate that there are six species present within the currently recognized species. There would probably be little dissent from scale insect taxonomists if we were to recognize and describe five additional species because the levels of genetic differentiation are high and the generally accepted tests of alternative species delimitation using Bayes Factors are decisive. However, as argued above, such application of species delimitation is arbitrary for parthenogenetic species and, given that *P*. *nigra* is considered a pest, the naming of new species could have implications for international trade.

In general, most insect herbivores are relatively host specific, feeding on plants of only one plant family or genus [21, 101–104] leading to the question of whether some polyphagous species are, in fact, multiple host-specific lineages that are morphologically cryptic. This has been found to be the case in some scale insects (e.g. [105–107]) and in other insects (e.g. [108–112]) in which host-specific lineages have been identified in what was originally considered to be a single species. Here, however, we found no evidence that *P*. *nigra* comprises host-specific lineages because clades are not host-specific and there was sharing of hosts across lineages in allopatry and sympatry. The lineages of *P*. *nigra* might be truly polyphagous, like some other generalist insects (e.g. [113–117]).

In Australia, there are two lineages that appear to be ecologically distinct: one in temperate regions and the other in subtropical regions. The ecological distinctiveness indicated by the niche identity test for Australian collections is supported by the geographical distribution of other members of those lineages: the lineage restricted to temperate regions of Australia (ANZ) occurs also in New Zealand, another temperate region, and the lineage shown to be subtropical in Australia (W3) occurs in some other tropical areas (Colombia, Thailand and Malaysia). The ecological distinctiveness of the two lineages in Australia (ANZ and W3) is striking, especially given that they overlap in latitude and longitude (Fig 3). At first glance, three members of W3 seem to be outliers in the species distribution model (Fig 3B), but these individuals were collected from highly human-modified and maintained environments (e.g. supermarket carpark and vineyard).

In some localities, multiple genetic lineages of *P*. *nigra* occur in sympatry, such as W1 and W2 in Taiwan, indicating no clear ecological distinction. All lineages except ANZ appear to occur in warm regions (subtropical and tropical) and there is no clear geographic structuring. Overall, there appears to be two ecotypes of *P*. *nigra*—temperate (ANZ) and subtropical/tropical (the rest). Only lineage ANZ appears to be ecologically differentiated from other lineages and it is therefore the only one that warrants consideration as a distinct species. The type collection of *P*. *nigra* was from Sri Lanka, a subtropical/tropical region, and thus the ANZ clade should be described and named as a new recognized taxon if taxonomic changes are to be made. With better geographic sampling, greater distinction might be discovered among the other genetic lineages that indicate ecological or phenotypic differentiation not evident here.

If lineage ANZ is to be recognized as a distinct species and the remaining lineages are to be kept under *P*. *nigra*, we will have a situation where ANZ is monophyletic but *P*. *nigra* is not (because ANZ is nested within). Paraphyly is probably inherent during early speciation in many sexual species [118] and among bacterial species [119], and a criterion of reciprocal monophyly should not be viewed as necessary for delimiting species [120–121], even though it might be favored for higher-level classifications. Unless ecological or phenotypic differences are found for the other main lineages of *P*. *nigra* (G, ICB and W1-3), we think it prudent to consider *P*. *nigra* as comprising at least two biotypes (ecotypes), one of which (ANZ) should probably be described and named as distinct species.

### Consequences for quarantine

A quarantine pest is a species, biotype or strain of plant, animal or pathogen that has the potential to cause economic or other damage in an area where it does not currently occur, and which is not widespread or being officially controlled already [122]. Because of the presumed cosmopolitan distribution of *P*. *nigra*, it does not attract significant attention from quarantine authorities and is not included in the quarantine strategies of most areas (e.g. [12, 22, 123]). We have shown that the currently recognized *P*. *nigra* is likely a species complex, with each lineage being polyphagous: there is potential for further cross-border incursions. However, given the limited sampling available from outside Australia, it is not known whether the ANZ lineage is more widespread than just Australia and New Zealand. In particular, we have no DNA sequences for specimens from temperate regions of the northern hemisphere. This is relevant because many of these countries import hosts of *P*. *nigra* (e.g. apples, citrus and grapes) from Australia and New Zealand [124–125] where lineage ANZ is present. More generally, a greater awareness of the existence of cryptic diversity within *P*. *nigra* is warranted.

## Supporting information

S1 Appendix Method of maximum parsimony (MP). (DOCX)

S2 Appendix The 19 environmental layers used in species distribution modelling analyses. (DOC)

S3 Appendix GenBank accession numbers of sequences used in this study. (DOC)

S1 Fig The maximum clade credibility (MCC) tree from Bayesian inferences using the concatenated dataset (2849 bp) and 71 specimens. Samples of the six clades of *Parasaissetia nigra* have been collapsed into triangles and the colors of clades are as shown in Fig 1. The colored squares around branches indicate that the branch was strongly supported (bootstrap values ≥ 70% in maximum parsimony and Bayesian posterior probabilities ≥ 0.95) in analyses of that dataset. Branch supports among outgroup taxa are not shown.
(EPS)

S2 Fig BEAST maximum clade credibility (MCC) tree inferred using *COI* dataset (939 bp) and 65 specimens of *Parasaissetia nigra*. The branch support values are indicated as Bayesian posterior probabilities, and only values ≥ 0.95 are shown. Specimens are color-coded by clade as shown in Fig 1. Abbreviations are the same as what used in Table 1.
(EPS)

S3 Fig BEAST maximum clade credibility (MCC) tree inferred using *Dynamin* dataset (599 bp) and 62 specimens of *Parasaissetia nigra*. The branch support values are indicated as Bayesian posterior probabilities, and only values ≥ 0.95 are shown. Specimens are color-coded by clade as shown in Fig 1. Abbreviations are the same as what used in Table 1.
(EPS)

S4 Fig BEAST maximum clade credibility (MCC) tree inferred using *EF-1α* dataset (620 bp) and 65 specimens of *Parasaissetia nigra*. The branch support values are indicated as Bayesian posterior probabilities, and only values ≥ 0.95 are shown. Specimens are color-coded by clade as shown in Fig 1. The monophyly of Ghana (G) clade is not supported. Abbreviations are the same as what used in Table 1.
(EPS)

S5 Fig BEAST maximum clade credibility (MCC) tree inferred using *18S* + *28S* dataset (1296 bp) and 62 specimens of *Parasaissetia nigra*. The branch support values are indicated as Bayesian posterior probabilities, and only values ≥ 0.95 are shown. Specimens are color-coded by clade as shown in Fig 1. Only the monophyly of two clades, ANZ and W1, are supported. Abbreviations are the same as what used in Table 1.
(EPS)

S6 Fig The GMYC gene tree inferred using *COI* dataset (939 bp) and 65 specimens of *Parasaissetia nigra*. The red branches on the tree represent the six species delimited by GMYC, labelled as ANZ, W1, ICB, G, W2 and W3. Abbreviations are the same as what used in Table 1.
(EPS)

## References

[1] Mayr E. Systematics and the origin of species. New York: Columbia University Press; 1942.

[2] Paterson HE. The recognition concept of species In: Vrba ES, editor. Species and speciation. Pretoria: Transvaal Museum; 1985 pp. 21–29.

[3] Dobzhansky T. Genetics and the origin of species. New York: Columbia University Press; 1937.

[4] Dobzhansky T. Mendelian populations and their evolution. Am Nat. 1950; 84: 401–418.

[5] Wiley EO. The evolutionary species concept reconsidered. Syst Biol. 1978; 27: 17–26.

[6] Mishler BD. The morphological, developmental, and phylogenetic basis of species concepts in bryophytes. Bryologist. 1985; 88: 207–214.

[7] Hajibabaei M, Janzen DH, Burns JM, Hallwachs W, Hebert PDN. DNA barcodes distinguish species of tropical Lepidoptera. Proc Natl Acad Sci USA. 2006; 103: 968–971. 10.1073/pnas.0510466103 16418261 PMC1327734

[8] Hebert PDN, Cywinska A, Ball SL, deWaard JR. Biological identifications through DNA barcodes. Proc R Soc Lond B Biol Sci. 2003; 270: 313–321. 10.1098/rspb.2002.2218 PMC1691236 12614582

[9] Fujita MK, Leache AD, Burbrink FT, McGuire JA, Moritz C. Coalescent-based species delimitation in an integrative taxonomy. Trends Ecol Evol. 2012; 27: 480–488. 10.1016/j.tree.2012.04.012 22633974

[10] Mishler BD, Donoghue MJ. Species concepts: a case for pluralism. Syst Zool. 1982; 31: 491–503.

[11] Frankham R, Ballou JD, Dudash MR, Eldridge MDB, Fenster CB, Lacy RC, et al Implications of different species concepts for conserving biodiversity. Biol Conserv. 2012; 153: 25–31.

[12] Maynard GV, Hamilton JG, Grimshaw JF. Quarantine—Phytosanitary, sanitary and incursion management: an Australian entomological perspective. Aust J Entomol. 2004; 43: 318–328.

[13] Armstrong KF, Ball SL. DNA barcodes for biosecurity: invasive species identification. Philos Trans R Soc Lond B Biol Sci.2005; 360: 1813–1823. 10.1098/rstb.2005.1713 16214740 PMC1609225

[14] Boykin LM, Armstrong KF, Kubatko L, De Barro P. Species delimitation and global biosecurity. Evol Bioinform. 2012; 8: 1–37. 10.4137/EBO.S8532 PMC3256992 22267902

[15] Fontaneto D, Herniou EA, Boschetti C, Caprioli M, Melone G, Ricci C, et al Independently evolving species in asexual bdelloid rotifers. PLoS Biol.2007; 5: 914–921. 10.1371/journal.pbio.0050087 PMC1828144 17373857

[16] Birky CW, Adams J, Gemmel M, Perry J. Using population genetic theory and DNA sequences for species detection and identification in asexual organisms. PLoS One. 2010; 5: e10609 10.1371/journal.pone.0010609 20498705 PMC2869354

[17] Birky CW, Ricci C, Melone G, Fontaneto D. Integrating DNA and morphological taxonomy to describe diversity in poorly studied microscopic animals: new species of the genus *Abrochtha* Bryce, 1910 (Rotifera: Bdelloidea: Philodinavidae). Zool J Linn Soc.2011; 161: 723–734.

[18] Schön I, Pinto RL, Halse S, Smith AJ, Martens K, Birky CW. Cryptic species in putative ancient asexual darwinulids (Crustacea, Ostracoda). PLoS One. 2012; 7: e39844 10.1371/journal.pone.0039844 22802945 PMC3389007

[19] De Queiroz K. 5 The general lineage concept of species, species criteria, and the process of speciation: a conceptual unification and terminological recommendations In: Howard DJ, Berlocher SH, editors. Endless forms: Species and speciation. New York: Oxford University Press; 1998 pp. 57–75.

[20] Ben-Dov Y. Coccidae. ScaleNet: A Data Base Of The Scale Insects Of The World; 2012. Accessed: http://www.sel.barc.usda.gov/scalenet/scalenet.htm.

[21] Lin Y-P, Cook DH, Gullan PJ, Cook LG. Does host-plant diversity explain species richness in insects? A test using Coccidae (Hemiptera). Ecol Entomol. 2015; 40: 299–306.

[22] Malumphy C. Diagnostic protocols for regulated pests *Parasaissetia nigra*. Bull OEPP. 2002; 32: 293–298.

[23] Kosztarab M. 3.3.13 Ornamental and house plants In: Ben-Dov Y, Hodgson CJ, editors. Soft scale insects: Their biology, natural enemies and control, volume 7B: World crop pest. Amsterdam: Elsevier Science BV; 1997 pp. 357–366.

[24] Hamon AB, Williams ML. The soft scale insects of Florida (Homoptera: Coccoidea: Coccidae). Gainesville: Florida Department of Agriculture & Consumer Services, Division of Plant Industry; 1984.

[25] Chua TH. 3.3.18 Rubber In: Ben-Dov Y, Hodgson CJ, editors. Soft scale insects: Their biology, natural enemies and control, volume 7B: World crop pest. Amsterdam: Elsevier Science BV; 1997 pp. 395–399

[26] Swirski E, Ben-Dov Y, Wysoki M. 3.3.7 Other subtropical fruit trees In: Ben-Dov Y, Hodgson CJ, editors. Soft scale insects: Their biology, natural enemies and control, volume 7B: World crop pest. Amsterdam: Elsevier Science BV; 1997 pp. 271–292.

[27] Shree MP, Manjunatha S. Incidence of black scale insects (*Saissetia nigra*, N.) infesting mulberry in Kanakapura taluk (Bangalore Rural District, Karnataka State). Entomon. 2000; 25: 91–96.

[28] Causton CE, Peckb SB, Sinclairc BJ, Roque-Albeloa L, Hodgson CJ, Landry B. Alien insects: threats and implications for conservation of Galápagos Islands. Ann Entomol Soc Am. 2006; 99: 121–143.

[29] Smith RH. Bionomics and control of the nigra scale, *Saissetia nigra*. Hilgardia. 1944; 16: 225–288.

[30] Ben-Dov Y. Taxonomy of the nigra scale *Parasaissetia nigra* (Nietner) (Homoptera: Coccoidea: Coccidae), with observations on mass rearing and parasites of an Israeli strain. Phytoparasitica. 1978; 6: 115–127.

[31] Gill RJ. The scale insects of California: Part 1. The soft scales (Homoptera: Coccoidea: Coccidae). Sacramento: California Department of Food & Agriculture; 1988.

[32] Green EE. The Coccidae of Ceylon—III. London: Dulau & Co; 1904.

[33] Miller GL. Morphology and systematics of the male tests and adult males of the family Coccidae (Homoptera: Coccoidea) from America north of Mexico. Ph.D. Thesis, Auburn University. 1991.

[34] Cook LG. *Apiomorpha gullanae* sp n., an unusual new species of gall-inducing scale insect (Hemiptera: Eriococcidae). Aust J Entomol. 2003; 42: 327–333.

[35] De Lotto G. The identity of some East African species of *Saissetia* (Homoptera, Coccidae). Bull Entomol Res. 1956; 47: 239–249.

[36] De Lotto G. The soft scales (Homoptera: Coccidae) of South Africa I. S Afr J Agric Sci. 1967; 10: 781–810.

[37] Hodgson CJ. The scale insect family Coccidae: An identification manual to genera. Wallingford: CAB International; 1994.

[38] Heled J, Drummond AJ. Bayesian inference of species trees from multilocus data. Mol Biol Evol. 2010; 27: 570–580. 10.1093/molbev/msp274 19906793 PMC2822290

[39] Yang ZH, Rannala B. Bayesian species delimitation using multilocus sequence data. Proc Natl Acad Sci USA. 2010; 107: 9264–9269. 10.1073/pnas.0913022107 20439743 PMC2889046

[40] Knowles LL, Carstens BC. Delimiting species without monophyletic gene trees. Syst Biol. 2007; 56: 887–895. 10.1080/10635150701701091 18027282

[41] Mallet J. A species definition for the modern synthesis. Trends Ecol Evol. 1995; 10: 294–299. 21237047 10.1016/0169-5347(95)90031-4

[42] Van Valen L. Ecological species, multispecies, and oaks. Taxon. 1976; 25: 233–239.

[43] Andersson L. The driving force: Species concepts and ecology. Taxon. 1990; 9: 375–382.

[44] Takahashi R. Key to the genera of Coccidae in Japan, with descriptions of two new genera and a little-known species (Homoptera). Insecta Matsumurana. 1955; 19: 23–28.

[45] Nietner J. Observations on the enemies of the coffee tree in Ceylon. Ceylon Times. 1861.

[46] Rosenblueth M, Sayavedra L, Sámano-Sánchez H, Roth A, Martínez-Romero E. Evolutionary relationships of flavobacterial and enterobacterial endosymbionts with their scale insect hosts (Hemiptera: Coccoidea). J Evol Biol. 2012; 25: 2357–2368. 10.1111/j.1420-9101.2012.02611.x 22994649

[47] Lin Y-P, Kondo T, Gullan PJ, Cook LG. Delimiting genera of scale insects: molecular and morphological evidence for synonymising *Taiwansaissetia* Tao, Wong and Chang with *Coccus* Linnaeus (Hemiptera: Coccoidea: Coccidae). Syst Entomol. 2013; 38: 249–264.

[48] Rakimov A, Ben-Dov Y, White V, Hoffmann AA. Soft scale insects (Hemiptera: Coccoidea: Coccidae) on grapevines in Australia. Aust J Entomol. 2013; 52: 371–378.

[49] Miller DR, Hodgson CJ. 1.1.3.7 Phylogeny In: Ben-Dov Y, Hodgson CJ, editors. Soft scale insects: Their biology, natural enemies and control, volume 7B: World crop pest. Amsterdam: Elsevier Science BV; 1997 pp. 229–250.

[50] Ben-Dov Y, Hodgson CJ. 1.4 Techniques In: Ben-Dov Y, Hodgson CJ, editors. Soft scale insects: Their biology, natural enemies and control, volume 7B: World crop pest. Amsterdam: Elsevier Science BV; 1997 pp. 389–395.

[51] Williams DJ, Watson GW. The scale insects of the tropical south Pacific region, part 3. The soft scales (Coccidae) and other families. Wallingford: CAB International; 1990.

[52] Hoy MA. Chapter 13 Insect molecular systematics and evolution In: Hoy MA, editor. Insect molecular genetics: An introduction to principles and applications. San Diego: Academic Press; 1994 pp. 337–387.

[53] Simon C, Frati F, Beckenbach A, Crespi B, Liu H, Flook P. Evolution, weighting and phylogenetic utility of mitochondrial gene sequences and a compilation conserved polymerase chain reaction primers. Ann Entomol Soc Am. 1994; 87: 651–701.

[54] Cho S, Mitchell A, Regier JC, Mitter C, Poole RW, Friedlander TP, et al A highly conserved nuclear gene for low-level phylogenetics: *Elongation Factor-1α* recovers morphology-based tree for heliothine moths. Mol Biol Evol. 1995; 12: 650–656. 7659020 10.1093/oxfordjournals.molbev.a040244

[55] Hardy NB. Phylogenetic utility of dynamin and triose phosphate isomerase. Syst Entomol. 2007; 32: 396–403.

[56] Dowton M, Austin AD. Phylogenetic relationships among the microgastroid wasps (Hymenoptera: Braconidae): Combined analysis of 16S and 28S rDNA genes and morphological data. Mol Phylogenet Evol. 1998; 10: 354–366. 10.1006/mpev.1998.0533 10051388

[57] Whiting MF, Carpenter JC, Wheeler QD, Wheeler WC. The Strepsiptera problem: Phylogeny of the holometabolous insect orders inferred from 18S and 28S ribosomal DNA sequences and morphology. Syst Biol. 1997; 46: 1–68. 11975347 10.1093/sysbio/46.1.1

[58] von Dohlen CD, Moran NA. Molecular phylogeny of the Homoptera: A paraphyletic taxon. J Mol Evol. 1995; 41: 211–223. 7666451 10.1007/BF00170675

[59] Park DS, Suh SJ, Oh HW, Hebert PDN. Recovery of the mitochondrial *COI* barcode region in diverse Hexapoda through tRNA-based primers. BMC Genomics. 2010; 11: 423 10.1186/1471-2164-11-423 20615258 PMC2996951

[60] Folmer O, Black M, Hoeh W, Lutz R, Vrijenhoek R. DNA primers for amplification of mitochondrial cytochrome *c* oxidase subunit I from diverse metazoan invertebrates. Mol Mar Biol Biotechnol. 1994; 3: 294–299. 7881515

[61] Brady SG, Gadau J, Ward PS. Systematics of the ant genus *Camponotus* (Hymenoptera: Formicidae): A preliminary analysis using data from the mitochondrial gene *cytochrome oxidase I* In: Austin AD, Dowton M, editors. Hymenoptera: Evolution, biodiversity and biological control. Melbourne: CSIRO Publishing; 2000 pp. 131–139.

[62] Hardy NB, Gullan PJ, Henderson RC, Cook LG. Relationships among felt scale insects (Hemiptera: Coccoidea: Eriococcidae) of southern beech, *Nothofagus* (Nothofagaceae), with the first descriptions of Australian species of the *Nothofagus*-feeding genus *Madarococcus* Hoy. Invertebr Syst. 2008; 22: 365–405.

[63] Tamura K, Peterson D, Peterson N, Stecher G, Nei M, Kumar S. MEGA5: Molecular evolutionary genetics analysis using maximum likelihood, evolutionary distance, and maximum parsimony methods. Mol Biol Evol. 2011; 28: 2731–2739. 10.1093/molbev/msr121 21546353 PMC3203626

[64] Rambaut A. Se-Al: Sequence alignment editor. 1998. http://tree.bio.ed.ac.uk/software/seal/.

[65] Rogers J, Wall R. A mechanism for RNA splicing. Proc Natl Acad Sci USA. 1980; 77: 1877–1879. 6246511 10.1073/pnas.77.4.1877 PMC348611

[66] Jermiin LS, Ho SYW, Ababneh F, Robinson J, Larkum AWD. The biasing effect of compositional heterogeneity on phylogenetic estimates may be underestimated. Syst Biol. 2004; 53: 638–643. 10.1080/10635150490468648 15371251

[67] Swofford DL. PAUP*. Phylogenetic Analysis Using Parsimony (*and other methods), version 4. Sunderland: Sinauer Associates; 2003.

[68] Ronquist F, Huelsenbeck JP. MRBAYES 3: Bayesian phylogenetic inference under mixed models. Bioinformatics. 2003; 19: 1572–1574. 12912839 10.1093/bioinformatics/btg180

[69] Nylander JAA. MrModeltest v2. Uppsala: Evolutionary Biology Center, Uppsala University; 2004.

[70] Tavaré S. Some probabilistic and statistical problems in the analysis of DNA sequences. Lectures Math Life Sci. 1986; 17: 57–86.

[71] Kimura M. A simple method for estimating evolutionary rates of base substitutions through comparative studies of nucleotide sequences. J Mol Evol. 1980; 16: 111–120. 7463489 10.1007/BF01731581

[72] Jukes TH, Cantor CR. Evolution of protein molecules. Mammalian Protein Metabolism. 1969; 3: 21–132.

[73] Pedersen AG. Bayesian phylogenetic analysis. 2007. http://www.cbs.dtu.dk/dtucourse/cookbooks/gorm/27615/bayes1.php.

[74] Kass RE, Raftery AE. Bayes factors. J Am Stat Assoc. 1995; 90: 773–795.

[75] Drummond A, Rambaut A. Analyzing BEAST output. 2006. http://beast.bio.ed.ac.uk/analysing-beast-output.

[76] Rambaut A, Drummond AJ. Tracer, v1.6. 2013. http://tree.bio.ed.ac.uk/software/tracer/.

[77] Drummond AJ, Suchard MA, Xie D, Rambaut A. Bayesian Phylogenetics with BEAUti and the BEAST 1.7. Mol Biol Evol. 2012; 29: 1969–1973. 10.1093/molbev/mss075 22367748 PMC3408070

[78] Hasegawa M, Kishino H, Yano TA. Dating of the human—ape splitting by a molecular clock of mitochondrial-DNA. J Mol Evol. 1985; 22: 160–174. 3934395 10.1007/BF02101694

[79] Yule GU. A mathematical theory of evolution, based on the conclusions of Dr. J.C. Willis, F.R.S. Philos Trans R Soc Lond B Biol Sci. 1924; 213: 21–87.

[80] Aldous DJ. Stochastic models and descriptive statistics for phylogenetic trees, from Yule to today. Stat Sci. 2001; 16: 23–34.

[81] Gernhard T. The conditioned reconstructed process. J Theor Biol. 2008; 253: 769–778. 10.1016/j.jtbi.2008.04.005 18538793

[82] Newton MA, Raftery AE. Approximate Bayesian inference with the weighted likelihood bootstrap. J R Stat Soc Series B Stat Methodol. 1994; 56: 3–48.

[83] Baele G, Lemey P, Bedford T, Rambaut A, Suchard MA, Alekseyenko AV. Improving the accuracy of demographic and molecular clock model comparison while accommodating phylogenetic uncertainty. Mol Biol Evol. 2012; 29: 2157–2167. 10.1093/molbev/mss084 22403239 PMC3424409

[84] Raftery A, Newton M, Satagopan J, Krivitsky P. Estimating the integrated likelihood via posterior simulation using the harmonic mean identity In: Bernardo JM, Bayarri MJ, Berger JO, editors. Bayesian statistics 8. New York: Oxford University Press; 2007 pp. 371–416.

[85] Pons J, Barraclough TG, Gomez-Zurita J, Cardoso A, Duran DP, Hazell S, et al Sequence-based species delimitation for the DNA taxonomy of undescribed insects. Syst Biol. 2006; 55: 595–609. 16967577 10.1080/10635150600852011

[86] Gamble T, Colli GR, Rodrigues MT, Werneck FP, Simons AM. Phylogeny and cryptic diversity in geckos (*Phyllopezus*; Phyllodactylidae; Gekkota) from South America’s open biomes. Mol Phylogenet Evol. 2012; 62: 943–953. 10.1016/j.ympev.2011.11.033 22182991

[87] Paradis E, Claude J, Strimmer K. APE: Analyses of phylogenetics and evolution in R language. Bioinformatics. 2004; 20: 289–290. 14734327 10.1093/bioinformatics/btg412

[88] Ezard T, Fujisawa, T, Barraclough TG. SPLITS: SPecies LImits by Threshold Statistics. 2009. http://rpackages.ianhowson.com/rforge/splits/.

[89] R Development Core Team. R: a language and environment for statistical computing. Vienna: R Foundation for Statistical Computing; 2010.

[90] Phillips SJ, Anderson RP, Schapire RE. Maximum entropy modeling of species geographic distributions. Ecol Modell. 2006; 190: 231–259.

[91] Hijmans RJ, Cameron SE, Parra JL, Jones PG, Jarvis A. Very high resolution interpolated climate surfaces for global land areas. Int J Climatol. 2005; 25: 1965–1978.

[92] Warren DL, Glor RE, Turelli M. ENMTools: a toolbox for comparative studies of environmental niche models. Ecography. 2010; 33: 607–611.

[93] Cotterill FP, Taylor PJ, Gippoliti S, Bishop JM, Groves CP. Why one century of phenetics is enough: response to "are there really twice as many bovid species as we thought? Syst Biol. 2014; 63: 819–832. 10.1093/sysbio/syu003 24415680

[94] Kingman JFC. On the genealogy of large populations. J Appl Probab. 1982; 19: 27–43.

[95] Sukumaran J, Knowles LL. Multispecies coalescent delimits structure, not species. PNAS. 2017; 114: 1607–1612. 10.1073/pnas.1607921114 28137871 PMC5320999

[96] Lohse K. Can mtDNA barcodes be used to delimit species? A response to Pons et al. (2006). Syst Biol. 2009; 58: 439–442. 10.1093/sysbio/syp039 20525596

[97] Nei M, Takahata M. Effective population size, genetic diversity and coalescence time in subdivided populations. J Mol Evol. 1993; 37: 240–244. 8230248 10.1007/BF00175500

[98] Rodriguero MS, Lanteri AA, Confalonieri VA. Speciation in the asexual realm: Is the parthenogenetic weevil *Naupactus cervinus* a complex of species *in statu nascendi*? Mol Phylogenet Evol. 2013; 68: 644–656. 10.1016/j.ympev.2013.04.011 23623993

[99] Fitzhugh K. Species as explanatory hypotheses: refinements and implications. Acta Biotheor. 2009; 57: 201–248. 10.1007/s10441-009-9071-3 19224376

[100] Sokal RR, Crovello TJ. The biological species concept: a critical evaluation. Am Nat. 1970; 104: 127–153.

[101] Strong DR, Lawton JH, Southwood SR. Insects on plants: Community patterns and mechanisms. Oxford: Blackwell Scientific Publications; 1984.

[102] van Nieukerken EJ. Systematics and phylogeny of Holarctic genera of Nepticulidae (Lepidoptera: Heteroneura: Monotrysia). Zool Verh Leiden. 1986; 236: 1–93.

[103] Schoonhoven LM, van Loon JJA, Dicke M. Insect-plant biology. New York: Oxford University Press; 2005.

[104] Lin Y-P, Gullan PJ, Cook LG. Species richness and host-plant diversity are positively correlated in Coccidae. Entomol Hell. 2010; 19: 90–98.

[105] Cook LG, Rowell DM. Genetic diversity, host-specificity and unusual phylogeography of a cryptic, host-associated species complex of gall-inducing scale insects. Ecol Entomol. 2007; 32: 506–515.

[106] Gwiazdowski RA, Vea IM, Andersen JC, Normark BB. Discovery of cryptic species among North American pine-feeding *Chionaspis* scale insects (Hemiptera: Diaspididae). Biol J Linn Soc Lond. 2011; 104: 47–62.

[107] Mills PJ, Cook LG. Rapid chromosomal evolution in a morphologically cryptic radiation. Mol Phylogenet Evol. 2014; 77: 126–135. 10.1016/j.ympev.2014.03.015 24680740

[108] Hebert PDN, Penton EH, Burns JM, Janzen DH, Hallwachs W. Ten species in one: DNA barcoding reveals cryptic species in the neotropical skipper butterfly *Astraptes fulgerator*. Proc Natl Acad Sci USA. 2004; 101: 14812–14817. 10.1073/pnas.0406166101 15465915 PMC522015

[109] Blair CP, Abrahamson WG, Jackman JA, Tyrrell L. Cryptic speciation and host-race formation in a purportedly generalist tumbling flower beetle. Evolution. 2005; 59: 304–316. 15807417

[110] Smith MA, Woodley NE, Janzen DH, Hallwachs W, Hebert PDN. DNA barcodes reveal cryptic host-specificity within the presumed polyphagous members of a genus of parasitoid flies (Diptera: Tachinidae). Proc Natl Acad Sci USA. 2006; 103: 3657–3662. 10.1073/pnas.0511318103 16505365 PMC1383497

[111] Condon MA, Adams DC, Bann D, Flaherty K, Gammons J, Johnson J, et al Uncovering tropical diversity: six sympatric cryptic species of *Blepharoneura* (Diptera: Tephritidae) in flowers of *Gurania spinulosa* (Cucurbitaceae) in eastern Ecuador. Biol J Linn Soc Lond. 2008; 93: 779–797.

[112] Malenke JR, Johnson KP, Clayton DH. Host specialization differentiates cryptic species of feather-feeding lice. Evolution. 2009; 63: 1427–1438. 10.1111/j.1558-5646.2009.00642.x 19187249

[113] Boykin LM, Shatters RG, Rosell RC, McKenzie CL, Bagnall RA, De Barro P. et al Global relationships of *Bemisia tabaci* (Hemiptera: Aleyrodidae) revealed using Bayesian analysis of mitochondrial COI DNA sequences. Mol Phylogenet Evol. 2007; 44: 1306–1319. 10.1016/j.ympev.2007.04.020 17627853

[114] Hulcr J, Miller SE, Setliff GP, Darrow K, Mueller ND, Hebert PDN et al DNA barcoding confirms polyphagy in a generalist moth, *Homona mermerodes* (Lepidoptera: Tortricidae). Mol Ecol Notes. 2007; 7: 549–557.

[115] Andersen JC, Gruwell ME, Morse GE, Normark BB. Cryptic diversity in the *Aspidiotus nerii* complex in Australia. Ann Entomol Soc Am. 2010; 103: 844–854.

[116] Gullan PJ, Kaydan MB, Hardy NB. Molecular phylogeny and species recognition in the mealybug genus *Ferrisia* Fullaway (Hemiptera: Pseudococcidae). Syst Entomol. 2010; 35: 329–339.

[117] Correa M, Aguirre C, Germain JF, Hinrichsen P, Zaviezo T, Malausa T, et al A new species of *Pseudococcus* (Hemiptera: Pseudococcidae) belonging to the *Pseudococcus maritimus* complex from Chile: molecular and morphological description. Zootaxa. 2011; 2926: 46–54.

[118] Omland KE, Baker JM, Peters JL. Genetic signatures of intermediate divergence: population history of Old and New World Holarctic ravens (*Corvus corax*). Mol Ecol. 2006; 15: 795–808. 10.1111/j.1365-294X.2005.02827.x 16499703

[119] Cohan FM. What are bacterial species? Annu Rev Microbiol. 2002; 56: 457–487. 10.1146/annurev.micro.56.012302.160634 12142474

[120] Rieseberg LH, Brouillet L. Are many plant species paraphyletic? Taxon. 1994; 43: 21–32.

[121] Crisp MD, Chandler GT. Paraphyletic species. Telopea. 1996; 6: 813–844.

[122] Food and Agriculture Organization (FAO). A glossary of phytosanitary terms. International standard for phytosanitary measures #5. Rome: Food and Agriculture Organization; 2002.

[123] European and Mediterranean Plant Protection Organization (EPPO). EPPO A1 and A2 Lists of pests recommended for regulation as quarantine pests. 2013. http://www.eppo.int/QUARANTINE/quarantine.htm.

[124] Australian Trade Commission. Trade, import and export. 2015. http://www.australia.gov.au/information-and-services/business-and-industry/trade-import-and-export.

[125] Statistics New Zealand. Goods and services trade by country: Year ended June 2015. 2015. http://www.stats.govt.nz/browse_for_stats/industry_sectors/imports_and_exports/GoodsServicesTradeCountry_HOTPYeJun15.aspx.

