# Supplementary material for: Species delimitation in asexual insects of economic importance: The case of black scale (Parasaissetia nigra), a cosmopolitan parthenogenetic pest scale insect
Source: PLoS One. 2017 May 1;12(5):e0175889. doi: 10.1371/journal.pone.0175889 (PMC5411049; doi:10.1371/journal.pone.0175889)
Supplement: S1 Appendix — (DOCX) [file pone.0175889.s001.docx]

**S1 Appendix. Method of maximum parsimony (MP).**

A heuristic search was implemented in PAUP* 4.0b10 using 1000 random addition starts and applying a TBR (tree bisection reconnection) branch-swapping algorithm with no “maxtrees” restrictions. All sites were weighted equally for the nrDNA data (*18S* + *28S*) but a separate weighting scheme for each codon position (first: second: third = 2: 3: 1) was applied to the protein coding regions because the proportion of synonymous changes in the third codon positions is greater than that in the first positions, and none of the substitutions at the second codon position are synonymous. The strict consensus option was used to summarize MP trees and 1000 bootstrap pseudo-replicates were performed to assess the support for each node using the same parameters as the heuristic search.
