# Supplementary material for: Species delimitation in asexual insects of economic importance: The case of black scale (Parasaissetia nigra), a cosmopolitan parthenogenetic pest scale insect
Source: PLoS One. 2017 May 1;12(5):e0175889. doi: 10.1371/journal.pone.0175889 (PMC5411049; doi:10.1371/journal.pone.0175889)
Supplement: S2 Appendix — (DOC) [file pone.0175889.s002.doc]

**S2 Appendix.** **The 19 environmental layers used in species distribution modelling analyses.**

| **Short name** | **Type** | **Description** |
| --- | --- | --- |
| bioclim_bio1* | Temperature | annual (mean) |
| bioclim_bio2* | Temperature | diurnal range (mean) |
| bioclim_bio3 | Temperature | isothermality |
| bioclim_bio4 | Temperature | seasonality |
| bioclim_bio5* | Temperature | warmest period (max) |
| bioclim_bio6 | Temperature | coldest period (min) |
| bioclim_bio7 | Temperature | annual range |
| bioclim_bio8* | Temperature | wettest quarter (mean) |
| bioclim_bio9 | Temperature | driest quarter (mean) |
| bioclim_bio10 | Temperature | warmest quarter (mean) |
| bioclim_bio11 | Temperature | coldest quarter (mean) |
| bioclim_bio12 | Precipitation | annual |
| bioclim_bio13 | Precipitation | wettest period |
| bioclim_bio14 | Precipitation | driest period |
| bioclim_bio15 | Precipitation | seasonality |
| bioclim_bio16 | Precipitation | wettest quarter |
| bioclim_bio17 | Precipitation | driest quarter |
| bioclim_bio18* | Precipitation | warmest quarter |
| bioclim_bio19* | Precipitation | coldest quarter |

*Asterisks indicate the six layers selected for identity test.
