# Supplementary material for: Species delimitation in asexual insects of economic importance: The case of black scale (Parasaissetia nigra), a cosmopolitan parthenogenetic pest scale insect
Source: PLoS One. 2017 May 1;12(5):e0175889. doi: 10.1371/journal.pone.0175889 (PMC5411049; doi:10.1371/journal.pone.0175889)
Supplement: S3 Appendix — (DOC) [file pone.0175889.s003.doc]

**S3 Appendix. GenBank accession numbers of sequences used in this study.**

| **Species and Code** | **GenBank accession no. (*18S*)** | **GenBank accession no. (*28S*)** | **GenBank accession no. (*COI*, Barcode)** | **GenBank accession no. (*COI*, JB)** | **GenBank accession no. (*Dynamin*)** | **GenBank accession no. (*EF-1α*)** |
| --- | --- | --- | --- | --- | --- | --- |
| *Parasaissetia nigra* | | | | | | |
| YPL00011 | KY927551 | KY927561 | KY927571 | KY927581 | KY949348 | KY949410 |
| YPL00019 | KY927552 | KY927562 | KY927572 | KY927582 | KY949349 | KY949411 |
| YPL00073 | KY927553 | KY927563 | KY927573 | KY927583 | KY949350 | KY949412 |
| YPL00075 | KY927554 | KY927564 | KY927574 | KY927584 | KY949351 | KY949413 |
| YPL00078 | KY927555 | KY927565 | KY927575 | KY927585 | KY949352 | KY949414 |
| YPL00080 | KY927556 | KY927566 | KY927576 | KY927586 | KY949353 | KY949415 |
| YPL00083 | KY927557 | KY927567 | KY927577 | KY927587 | KY949354 | KY949416 |
| YPL00085 | KY927558 | KY927568 | KY927578 | KY927588 | KY949355 | KY949417 |
| YPL00089 | KY927559 | KY927569 | KY927579 | KY927589 | KY949356 | KY949418 |
| YPL00099 | KY927560 | KY927570 | KY927580 | KY927590 | KY949357 | KY949419 |
| YPL00118 | KY927591 | KY927601 | KY927611 | KY927621 | KY949358 | KY949420 |
| YPL00119 | KY927592 | KY927602 | KY927612 | KY927622 | KY949359 | KY949421 |
| YPL00126 | KY927593 | KY927603 | KY927613 | KY927623 | KY949360 | KY949422 |
| YPL00238 | KY927594 | KY927604 | KY927614 | KY927624 | KY949361 | KY949423 |
| YPL00239 | KY927595 | KY927605 | KY927615 | KY927625 | KY949362 | KY949424 |
| YPL00243 | KY927596 | KY927606 | KY927616 | KY927626 | KY949363 | KY949425 |
| YPL00256 | KY927597 | KY927607 | KY927617 | KY927627 | KY949364 | KY949426 |
| YPL00260 | KY927598 | KY927608 | KY927618 | KY927628 | KY949365 | KY949427 |
| YPL00284 | KY927599 | KY927609 | KY927619 | KY927629 | KY949366 | KY949428 |
| YPL00287 | KY927600 | KY927610 | KY927620 | KY927630 | KY949367 | KY949429 |
| YPL00289 | KY927631 | KY927641 | KY927651 | KY927661 | KY949401 | KY949435 |
| YPL00315 | KY927632 | KY927642 | KY927652 | KY927662 | KY949402 | KY949436 |
| YPL00323 | KY927633 | KY927643 | KY927653 | KY927663 | KY949403 | KY949437 |
| YPL00337 | KY927634 | KY927644 | KY927654 | KY927664 | KY949404 | KY949438 |
| YPL00340 | KY927635 | KY927645 | KY927655 | KY927665 | n.a. | KY949439 |
| YPL00356 | KY927636 | KY927646 | KY927656 | KY927666 | KY949405 | KY949440 |
| YPL00361 | KY927637 | KY927647 | KY927657 | KY927667 | KY949406 | KY949441 |
| YPL00364 | KY927638 | KY927648 | KY927658 | KY927668 | KY949407 | KY949442 |
| YPL00426 | KY927639 | KY927649 | KY927659 | KY927669 | KY949408 | KY949443 |
| YPL00449 | KY927640 | KY927650 | KY927660 | KY927670 | KY949409 | KY949444 |
| YPL00462 | KY927671 | KY927681 | KY927691 | KY927701 | KY949381 | KY949445 |
| YPL00473 | KY927672 | KY927682 | KY927692 | KY927702 | KY949382 | KY949446 |
| YPL00474 | KY927673 | KY927683 | KY927693 | KY927703 | KY949383 | KY949447 |
| YPL00476 | KY927674 | KY927684 | KY927694 | KY927704 | KY949384 | KY949448 |
| YPL00477 | KY927675 | KY927685 | KY927695 | KY927705 | KY949385 | KY949449 |
| YPL00478 | KY927676 | KY927686 | KY927696 | KY927706 | KY949386 | KY949450 |
| YPL00483 | KY927677 | KY927687 | KY927697 | KY927707 | KY949387 | KY949451 |
| YPL00487 | KY927678 | KY927688 | KY927698 | KY927708 | KY949388 | KY949452 |
| YPL00492 | KY927679 | KY927689 | KY927699 | KY927709 | KY949389 | KY949453 |
| YPL00495 | KY927680 | KY927690 | KY927700 | KY927710 | KY949390 | KY949454 |
| YPL00498 | KY933311 | **KY933321** | KY933331 | KY933341 | KY949391 | KY949455 |
| YPL00499 | KY933312 | **KY933322** | KY933332 | KY933342 | KY949392 | KY949456 |
| YPL00500 | KY933313 | **KY933323** | KY933333 | KY933343 | KY949393 | KY949457 |
| YPL00522 | KY933314 | **KY933324** | KY933334 | KY933344 | KY949394 | KY949458 |
| YPL00525 | KY933315 | **KY933325** | KY933335 | KY933345 | KY949395 | KY949459 |
| YPL00540 | KY933316 | **KY933326** | KY933336 | KY933346 | KY949396 | KY949460 |
| YPL00544 | KY933317 | **KY933327** | KY933337 | KY933347 | KY949397 | KY949461 |
| YPL00548 | KY933318 | **KY933328** | KY933338 | KY933348 | KY949398 | KY949462 |
| YPL00551 | KY933319 | **KY933329** | KY933339 | KY933349 | KY949399 | KY949463 |
| YPL00556 | KY933320 | **KY933330** | KY933340 | KY933350 | KY949400 | KY949464 |
| YPL00562 | KY933351 | KY933361 | KY933371 | KY933381 | n.a. | KY949465 |
| YPL00573 | KY933352 | KY933362 | KY933372 | KY933382 | KY949372 | KY949466 |
| YPL00574 | KY933353 | KY933363 | KY933373 | KY933383 | KY949373 | KY949467 |
| YPL00578 | KY933354 | KY933364 | KY933374 | KY933384 | KY949374 | KY949468 |
| YPL00620 | KY933355 | KY933365 | KY933375 | KY933385 | KY949375 | KY949469 |
| YPL00688 | KY933356 | KY933366 | KY933376 | KY933386 | KY949376 | KY949470 |
| YPL00692 | KY933357 | KY933367 | KY933377 | KY933387 | KY949377 | KY949471 |
| YPL00697 | KY933358 | KY933368 | KY933378 | KY933388 | KY949378 | KY949472 |
| YPL00699 | KY933359 | KY933369 | KY933379 | KY933389 | KY949379 | KY949473 |
| YPL00723 | KY933360 | KY933370 | KY933380 | KY933390 | KY949380 | KY949474 |
| YPL00734 | KY938538 | KY938543 | KY938548 | KY938553 | KY949368 | KY949430 |
| TK0151 | KY938539 | KY938544 | KY938549 | KY938554 | KY949369 | KY949431 |
| TK0177 | KY938540 | KY938545 | KY938550 | KY938555 | KY949370 | KY949432 |
| TK0187 | KY938541 | KY938546 | KY938551 | KY938556 | KY949371 | KY949433 |
| TK0205 | KY938542 | KY938547 | KY938552 | KY938557 | n.a. | KY949434 |
| *Coccus hesperidum* | | | | | | |
| YPL00076 | JX566902 | JX627324 | JX843722 | KY927541 | KY934479 | JX945995 |
| *C*. *longulus* | | | | | | |
| YPL00433 | JX853919 | JX866693 | JX853907 | KY927542 | KY934480 | JX965090 |
| *Saissetia* *coffeae* | | | | | | |
| YPL00104 | JX566917 | JX645353 | JX845480 | KY927543 | KY934481 | JX965091 |
| *S. miranda* | | | | | | |
| YPL00032 | JX866682 | JX866694 | JX853908 | KY927544 | KY934482 | JX965101 |
| *S*. *oleae* | | | | | | |
| YPL00246 | KY927547 | KY927549 | KY927539 | KY927545 | KY934483 | KY938536 |
| *S*. *somereni* | | | | | | |
| YPL00237 | KY927548 | KY927550 | KY927540 | KY927546 | KY934484 | KY938537 |
